# Comparative Lysine Acetylome Analysis of Y. pestis YfiQ/CobB Mutants Reveals that Acetylation of SlyA Lys73 Significantly Promotes Biofilm Formation of Y. pestis

**DOI:** 10.1128/spectrum.00460-23

**Published:** 2023-07-17

**Authors:** Yafang Tan, Wanbing Liu, Yuling Chen, Yazhou Zhou, Kai Song, Shiyang Cao, Yuan Zhang, Yajun Song, Haiteng Deng, Ruifu Yang, Zongmin Du

**Affiliations:** a State Key Laboratory of Pathogen and Biosecurity, Beijing Institute of Microbiology and Epidemiology, Beijing, China; b MOE Key Laboratory of Bioinformatics, School of Life Sciences, Tsinghua University, Beijing, China; Navarrabiomed-Universidad Pública de Navarra (UPNA)-Complejo Hospitalario de Navarra (CHN), IdiSNA

**Keywords:** lysine acetylation, posttranslational modifications (PTMs), mass spectrometry (MS), *Yersinia pestis*, biofilms

## Abstract

Increasing evidence shows that protein lysine acetylation is involved in almost every aspect of cellular physiology in bacteria. Yersinia pestis is a flea-borne pathogen responsible for millions of human deaths in three global pandemics. However, the functional role of lysine acetylation in this pathogen remains unclear. Here, we found more acetylated proteins and a higher degree of acetylation in Y. pestis grown under mammalian host (Mh) conditions than under flea vector (Fv) conditions, suggesting that protein acetylation could significantly change during fleabite transmission. Comparative acetylome analysis of mutants of YfiQ and CobB, the major acetyltransferase and deacetylase of Y. pestis, respectively, identified 23 YfiQ-dependent and 315 CobB-dependent acetylated proteins. Further results demonstrated that acetylation of Lys73 of the SlyA protein, a MarR-family transcriptional regulator, inhibits its binding to the promoter of target genes, including *hmsT* that encodes diguanylate cyclase responsible for the synthesis of c-di-GMP, and significantly enhances biofilm formation of Y. pestis. Our study presents the first extensive acetylome data of Y. pestis and a critical resource for the functional study of lysine acetylation in this pathogen.

**IMPORTANCE** Yersinia pestis is the etiological agent of plague, historically responsible for three global pandemics. The 2017 plague epidemic in Madagascar was a reminder that Y. pestis remains a real threat in many parts of the world. Plague is a zoonotic disease that primarily infects rodents via fleabite, and transmission of Y. pestis from infected fleas to mammals requires rapid adaptive responses to adverse host environments to establish infection. Our study provides the first global profiling of lysine acetylation derived from mass spectrometry analysis in Y. pestis. Our data set can serve as a critical resource for the functional study of lysine acetylation in Y. pestis and provides new molecular insight into the physiological role of lysine acetylation in proteins. More importantly, we found that acetylation of Lys73 of SlyA significantly promotes biofilm formation of Y. pestis, indicating that bacteria can use lysine acetylation to fine-tune the expression of genes to improve adaptation.

## INTRODUCTION

Yersinia pestis is the causative agent of plague, known historically as the notorious Black Death (1). Records of plague go back as far as 2,000 years ago, and it has caused three global pandemics (2). Although plague epidemics have been generally well controlled in recent decades, natural foci of plague still exist in Africa, Asia, and the Americas, and sporadic outbreaks of plague remain a serious threat to public health (3). Further, Y. pestis is a select agent with bioterrorism potential (4).

Since the discovery of protein acetylation over 50 years ago, most studies have focused primarily on histones and other transcription-associated proteins (5). The discovery of nonhistone acetylated proteins provides a new perspective for understanding protein acetylation. Acetylation can happen both co- and posttranslationally on the α-amino group at the N terminus of the protein (“N-terminal acetylation”) or on the ε-amino group on the side chain of lysine (*N*^ε^-acetylation), serine, and threonine (*O*-acetylation) (6). In addition to its role in transcriptional regulation, lysine acetylation (LysAc) affects many biological processes, including central metabolism, protein synthesis and degradation, cell morphology, cell cycle regulation, and apoptosis (7–10). Acetylation modification may play a biological function as important as phosphorylation (11). Protein acetylation was previously considered to occur predominantly in eukaryotes; however, advances in mass spectrometry (MS)-based proteomics and high-affinity enrichment of acetylated peptides have revealed that protein acetylation is a widespread posttranslational modification (PTM) that occurs across all domains of life (12–14). Recently, acetylation and deacetylation of some proteins has been reported as related to the virulence of bacteria (15).

Lysine acetylation is a dynamic and reversible process catalyzed by acetyltransferase and deacetylase, in which an acetyl group is transferred between an acetyl donor, such as acetyl-coenzyme A (Ac-CoA), and the amino group of a protein (16). In our previous study, we identified YfiQ and CobB, encoded by *yp_1760* and *yp_0659* according to the genome annotation of Y. pestis strain 91001 (17, 18), as the major acetyltransferase and deacetylase responsible for protein acetylation modification in Y. pestis. Mutation of the *yfiQ* or *cobB* genes leads to defects in stress responses to environmental conditions, including acidic pH, cold and heat shock, and oxidative stress (19). YfiQ is the most intensively studied acetyltransferase in prokaryotes. As a Gcn-5-like acetyltransferase, it catalyzes the transfer of an acetyl group from Ac-CoA to the ε position of lysine (*N*^ε^-Lys) (20). CobB is a deacetylase belonging to the NAD^+^-dependent sirtuin family and is highly conserved among prokaryotes (21, 22). In nature, Y. pestis is predominately transmitted among rodents through fleabites and occasionally from fleas to humans, causing human plague. After entering the host, the bacteria are readily taken up by host professional phagocytes, and those engulfed by macrophages can survive and replicate in this shield niche (23, 24). Transition between the two drastically different environments of flea vector (Fv) and mammalian host (Mh) demands a rapid adaptation of Y. pestis to external conditions, especially those in the Mh, to avoid being eliminated before establishing a successful infection.

To investigate the role of protein acetylation in adaptation to the two typical niches of Y. pestis Mh and Fv, comparative acetylome analyses of the wild-type (WT) and *yfiQ* or *cobB* mutants of Y. pestis were performed. In total, 1,397 acetylated proteins were detected, accounting for 32.6% of all proteins, among which 23 were YfiQ-dependent acetylated proteins and 315 were CobB-dependent acetylated proteins. Furthermore, we found that the acetylation states of Lys73 in the SlyA protein, a member of the multiple antibiotic resistance regulator (MarR) transcription factor family associated with bacterial responses to host-derived oxidative stress, antibiotic resistance, biofilm formation, and virulence (25–27), might be regulated by CobB deacetylase, and acetylation of Lys73 inhibited its DNA-binding ability and significantly enhanced biofilm formation of Y. pestis.

## RESULTS

### Comparative lysine acetylome analysis reveals that protein acetylation differs significantly in Y. pestis grown under conditions mimicking the two typical natural niches.

**FIG 1:**
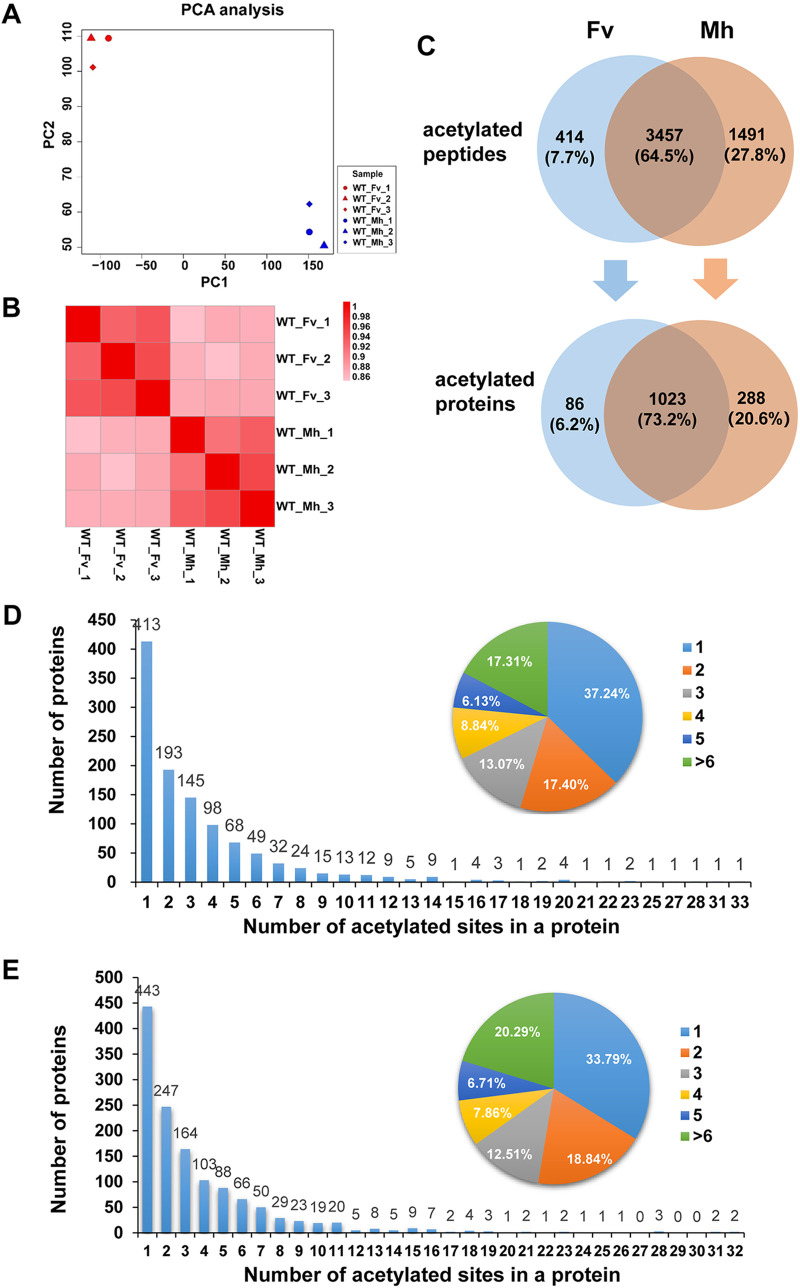
Global acetyl-proteomics analysis of Y. pestis grown under the Fv or Mh condition. (A and B) Reproducibility of the quantification measurements of Y. pestis grown under the Fv or Mh condition between three replicates shown with a PCA scatterplot (A) and a heatmap of correlation coefficients (B). (C) Venn diagram of the acetylated peptides (top) or proteins (bottom) that have been identified in Y. pestis cultured under the Fv or Mh condition. (D and E) Numbers of acetylated proteins containing different numbers of acetylated sites were plotted, and the proportion of proteins containing 1, 2, 3, 4, 5, and more than 6 acetylated sites were shown in pie charts for Y. pestis cultured under the Fv (D) or Mh (E) condition.

Acetylation modification is a highly reversible and dynamic process that gives rise to difficulties in the study of acetylomes (28). To identify as many lysine-acetylated proteins as possible, we analyzed the acetyl-proteomes of Y. pestis bacteria cultured under Mh and Fv conditions in three biological replicates. Total proteins were extracted in triplicate samples from the WT strain and two mutants (Δ*cobB* and Δ*yfiQ*). Each protein sample (40 μg) extracted was separated in a 4 to 15% SDS-PAGE gradient gel, and results showed that sample quality was comparable and suitable for MS analysis (Fig. S1 in the supplemental material). Acetylated peptides enriched by immunoprecipitation were analyzed by liquid chromatography-tandem MS (LC-MS/MS), and the results were searched using the MaxQuant search algorithm in the Y. pestis database. As shown in Table S1, 2,142, 2,987, and 2,811 acetylation sites were detected in the three replicates under the Fv condition. Among them, 1,471 (38%) acetylated peptides were detected in all three replicates, and 2,598 (67%) were detected at least twice. Similar results were obtained for the three replicates under the Mh condition. Principal-component analysis (PCA) (29, 30) was performed, and heatmaps of correlation coefficients (31) were drawn using R software (version 4.2.1) (32) for the data from the three replicates from the different groups (Fig. 1A and B). The results showed that the reproducibility and correlation coefficients between replicates of the same sample group were high, and the quantification results were reliable. Venn diagrams of the acetylated peptides detected in the three replicates cultured under each condition are shown in Fig. S2. More than half of the acetylated proteins were detected in all three replicates of Y. pestis grown under both conditions.

A total of 5,362 acetylated sites belonging to 1,397 acetylated proteins were detected in the 201 strain in three biological replicates, accounting for 32.6% of the total proteins encoded (1,397/4,280) (Fig. 1C). This indicates that acetylation is a frequently occurring modification in Y. pestis. As shown in Fig. 1C, a total of 3,457 acetylated peptides were detected in bacteria under both conditions, and 1,491 (27.8%) and 414 (7.7%) acetylated peptides were specific to bacteria cultured under Mh and Fv conditions, respectively. In addition to 1,023 acetylated proteins present in bacteria under both culture conditions, 288 (20.6%) acetylated proteins were specific to bacteria cultured under the Mh condition, significantly more than 86 (6.2%) acetylated proteins detected only under the Fv condition (Fig. 1C). These data indicate that more acetylated proteins were detected in Y. pestis grown under the Mh condition than under the Fv condition. Thus, Y. pestis appears to control the acetylation of a larger number of proteins when adapting to changing environmental conditions, including adverse Mh environments at 37°C, to promote infection.

### Proteins containing multiple lysine-acetylated sites are common in Y. pestis.

Many acetylated proteins have more than one acetylated site under both the Mh and Fv conditions. Among the 1,109 acetylated proteins detected in Y. pestis cultured under the Fv condition, approximately 37.24% contained only one acetylated site, 17.4% contained two acetylated sites, and 45.36% contained three or more acetylated sites (referred to here as “highly acetylated”). Interestingly, 17.31% of acetylated proteins contained more than six acetylated sites (Fig. 1D). Of the 1,311 acetylated proteins detected in Y. pestis cultured under the Mh condition, 33.79% had one acetylated site, 18.84% had two acetylated sites, 47.37% were highly acetylated, and 20.29% had more than six acetylated sites (Fig. 1E). Protein acetylation levels in Y. pestis grown under the Mh condition were higher than those under the Fv condition, suggesting that Y. pestis could regulate the acetylation of proteins to adapt to hostile environments after invasion into the mammalian host.

The top five Y. pestis proteins with the most acetylated sites were DnaK (33), Ymt (31), GroEL (28), AdhE (27), and RpoB (25) under the Fv condition and AdhE (33), DnaK (33), RpoB (32), Ymt (32), and GroEL (29) under the Mh condition. Among them, the chaperonin GroEL contains multiple acetylation sites in many bacteria, including Streptococcus *thermophiles* (12) (33), Mycobacterium tuberculosis (13) (14), and Pseudomonas aeruginosa (13) (34). This high lysine acetylation (LysAc) level has not been given a clear explanation, but it may reflect the involvement of LysAc in complex regulatory mechanisms that control the function or interactions of these proteins (35).

**FIG 2:**
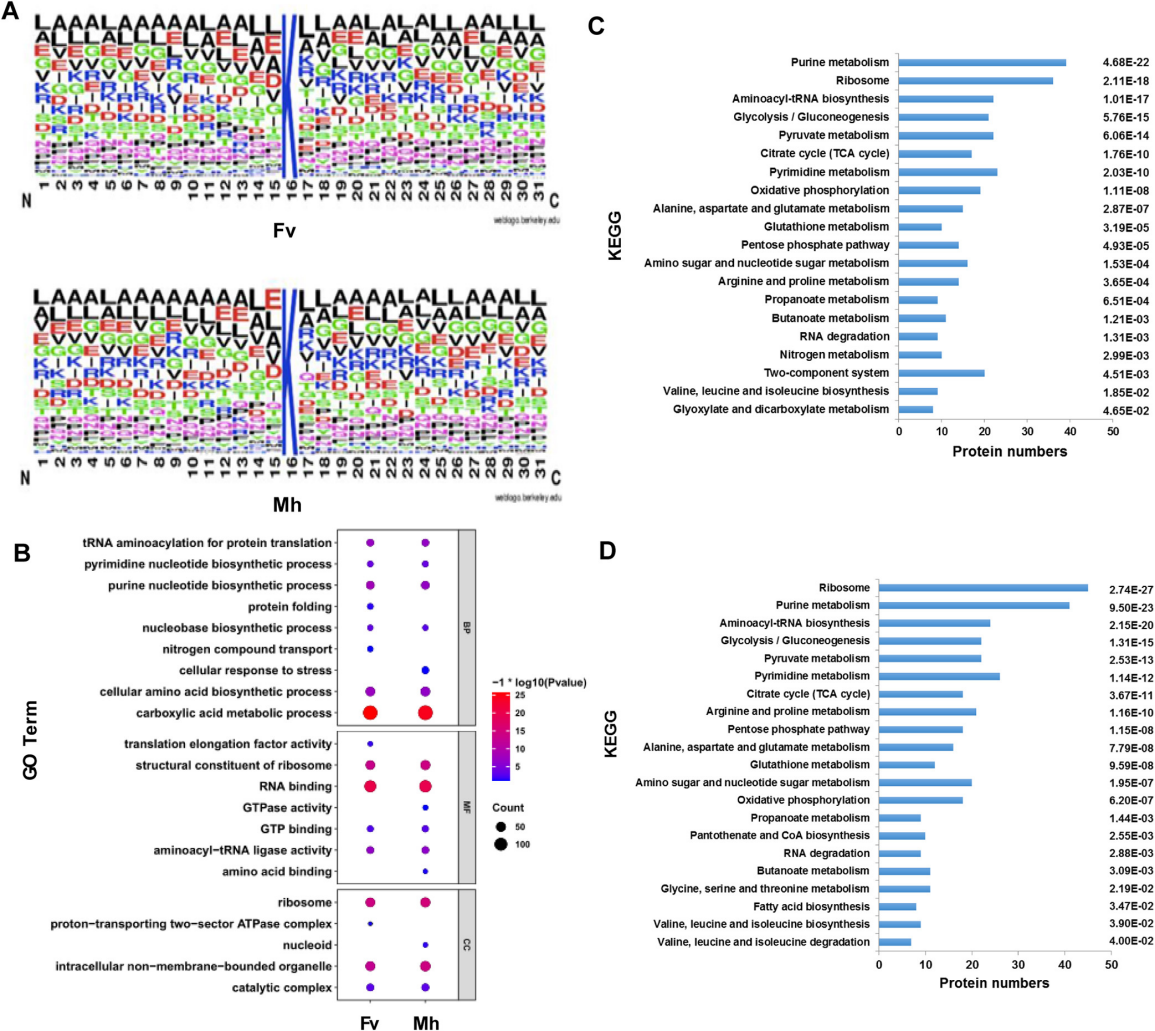
Analysis of lysine acetylation sites and functional cluster analysis of acetylated proteins in the Y. pestis 201 strain. (A) Web Logo was used to analyze the 30 amino acids flanking the acetylated sites detected in bacteria cultured under the Fv (top) or Mh (bottom) condition. (B) GO term and KEGG pathway enrichment analysis of acetylated proteins listed in the acetylome under Fv and Mh conditions. The dot plot shows the significance of the −log_10_ (*P* values) (color) and the number of proteins (size) in the enriched GO terms. (C and D) Bar plots showing the number of proteins (length) in the enriched KEGG pathways of the acetylated proteins detected in the bacteria cultured under the Fv (C) or Mh (D) condition.

To identify the potential consensus motifs for the acetylated sites, WebLogo software was used to analyze the occupancy frequency of amino acid residues flanking the acetylated sites in Y. pestis. Slight preferences for leucine or glutamic acid at the −1 position and for a leucine residue at the +1 position were observed in the bacteria cultured under both conditions (Fig. 2A).

### Functional analysis of acetylated proteins found that proteins acetylated only in Y. pestis grown under Mh conditions are involved in cellular response to stress.

To further explore the effects of acetylated proteins on the physiological function of Y. pestis, functional classification and enrichment analyses were performed. Gene Ontology (GO) term analysis was performed using web-based PANTHER bioinformatics tools (http://www.pantherdb.org/) (36), and enrichment significance was tested by Fisher’s exact test. The GO terms of carboxylic acid metabolic processes and biosynthesis processes, including those for amino acid, nucleobase, and RNA binding, were significantly enriched under both culture conditions (Fig. 2B). Some acetylated proteins detected only in bacteria cultured under the Fv condition were related to nitrogen compound transport, protein folding, and translation elongation factor activity, indicating that protein acetylation likely plays an important role in the regulation of protein synthesis (Table 1). The acetylated proteins detected only in bacteria cultured under the Mh condition were involved in cellular response to stress, including UvrA (a member of the UvrABC system that recognizes and repairs damaged DNA); the DNA mismatch repair protein MutS; RNase T, the enzyme that hydrolyzes the phosphodiester bond of RNA; a transcription factor repressor (LexA); the DNA strand-pairing protein RecA; a probable Fe^2+^-trafficking protein, and a catalase-peroxidase katG (Table 2). These proteins are critical for DNA mismatch repair, damaged DNA repair, and resistance to oxidative stress induced by external stimuli, suggesting that those functions could be activated by protein acetylation to improve the survival of Y. pestis in the adverse environment of the Mh.

**TABLE 1:** GO term enrichment analysis of acetylated proteins detected only in bacteria cultured under the Fv condition

GO terms	Gene ID	Gene name	Functional annotation
Protein folding (GO:0006457)	YP_2797	*skp*	Chaperone protein Skp
	YP_0775	*clpX*	ATP-dependent Clp protease ATP-binding subunit ClpX
	YP_3712	*dnaK*	Chaperone protein DnaK
	YP_0656	*clpB*	Chaperone protein ClpB
	YP_3711	*dnaJ*	Chaperone protein DnaJ
	YP_0505	*groS*	10 kDa chaperonin
	YP_2562	*hscB*	Cochaperone protein HscB
	YP_0506	*groL*	60 kDa chaperonin
	YP_0140	*hslO*	33 kDa chaperonin
	YP_1049	*grpE*	Protein GrpE
	YP_2563	*hscA*	Chaperone protein HscA
	YP_0811	*htpG*	Chaperone protein HtpG
	YP_0773	*tig*	Trigger factor
	YP_2469	*ureE*	Urease accessory protein UreE
Nitrogen compound transport (GO:0071705)	YP_3271	*tatA*	Sec-independent protein translocase protein TatA
	YP_3620	*secA*	Protein translocase subunit SecA
	YP_pCD36	*yscU*	Yop proteins translocation protein U
	YP_pCD32	*yscB*	Chaperone protein YscB
	YP_1032	*tolB*	Tol-Pal system protein TolB
	YP_2776	*metN1*	Methionine import ATP-binding protein MetN 1
	YP_4009	*yidC*	Membrane protein insertase YidC
	YP_0773	*tig*	Trigger factor
	YP_3272	*tatB*	Sec-independent protein translocase protein TatB
	YP_pCD40	*yscQ*	Yop proteins translocation protein Q
Translation elongation factor activity (GO:0003746)	YP_3117	*tufB*	Elongation factor Tu-B
	YP_1307		Elongation factor P-like protein
	YP_0201	*fusA*	Elongation factor G
	YP_0136	*greB*	Transcription elongation factor GreB
	YP_0578	*greA*	Transcription elongation factor GreA
	YP_2806	*tsf*	Elongation factor Ts
	YP_2520	*lepA*	Elongation factor 4
	YP_0202	*tufA*	Elongation factor Tu-A
Proton-transporting two-sector ATPase complex, catalytic domain (GO:0033178)	YP_4028	*atpD*	ATP synthase subunit beta
	YP_4029	*atpG*	ATP synthase gamma chain
	YP_4031	*atpH*	ATP synthase subunit delta
	YP_4027	*atpC*	ATP synthase epsilon chain
	YP_4030	*atpA*	ATP synthase subunit alpha

**TABLE 2:** GO term enrichment analysis of acetylated proteins detected only in bacteria cultured under the Mh condition

GO terms	Gene ID	Gene name	Functional annotation
Cellular response to stress (GO:0033554)	YP_0367	*katG*	Catalase-peroxidase
	YP_0480	*ssb*	Single-stranded DNA-binding protein
	YP_2168	*rnt*	Ribonuclease T
	YP_0479	*uvrA*	UvrABC system protein A
	YP_0333	*mutS*	DNA mismatch repair protein MutS
	YP_0470	*lexA*	LexA repressor
	YP_1899	*ruvC*	Crossover junction endodeoxyribonuclease RuvC
	YP_0656	*clpB*	Chaperone protein ClpB
	YP_0558	*msrA*	Peptide methionine sulfoxide reductase MsrA
	YP_2614	*ligA*	DNA ligase
	YP_0811	*htpG*	Chaperone protein HtpG
	YP_0379	*recA*	Protein RecA
	YP_1003	*uvrB*	UvrABC system protein B
	YP_2508	*ung*	Uracil-DNA glycosylase
	YP_3488		Probable Fe^2+^-trafficking protein
	YP_0527	*mutL*	DNA mismatch repair protein MutL
	YP_2524	*recO*	DNA repair protein RecO
	YP_4003	*recF*	DNA replication and repair protein RecF
	YP_0158	*gph*	Phosphoglycolate phosphatase
	YP_2547	*hmp*	Flavohemoprotein
	YP_1286	*nfo*	Probable endonuclease 4
	YP_1901	*ruvB*	Holliday junction ATP-dependent DNA helicase RuvB
Amino acid binding (GO:0016597)	YP_0381	*alaS*	Alanine-tRNA ligase
	YP_0638	*argI*	Ornithine carbamoyl transferase
	YP_3602	*gcvP*	Glycine dehydrogenase (decarboxylating)
	YP_2548	*glyA*	Serine hydroxymethyltransferase
	YP_3124	*argB*	Acetylglutamate kinase
	YP_0566	*argR*	Arginine repressor
	YP_3842	*pyrB*	Aspartate carbamoyl transferase
GTPase activity (GO:0003924)	YP_3117	*tufB*	Elongation factor Tu-B
	YP_2471	*ureG*	Urease accessory protein UreG
	YP_0520	*rsgA*	Small ribosomal subunit biogenesis GTPase RsgA
	YP_3751	*prfC*	Peptide chain release factor 3
	YP_0201	*fusA*	Elongation factor G
	YP_0574	*obg*	GTPase Obg
	YP_2520	*lepA*	Elongation factor 4
	YP_2523	*era*	GTPase Era
	YP_0202	*tufA*	Elongation factor Tu-A
	YP_0587	*infB*	Translation initiation factor IF-2
Nucleoid (GO:0009295)	YP_0480	*ssb*	Single-stranded DNA-binding protein
	YP_1189	*mukE*	Chromosome partition protein MukE
	YP_1188	*mukB*	Chromosome partition protein
	YP_1190	*mukF*	Chromosome partition protein
	YP_3638	*mraZ*	Transcriptional regulator MraZ
	YP_2524	*recO*	DNA repair protein RecO
	YP_0808		Nucleoid-associated protein YPO3121/y1061/YP_0808
	YP_0879		Nucleoid-associated protein YPO1262/y2922/YP_0879

KEGG pathway enrichment analyses showed that acetylation occurs on many proteins involved in carbohydrate metabolism (glycolysis/gluconeogenesis, pyruvate metabolism, citrate cycle, and the pentose phosphate pathway), nucleotide metabolism (purine metabolism and pyrimidine metabolism), amino acid metabolism, lipid metabolism, energy metabolism, RNA degradation, and aminoacyl-tRNA biosynthesis in bacteria cultured under both the Fv (Fig. 2C) and Mh (Fig. 2D) conditions. Acetylated proteins can apparently participate in various fundamental physiological functions, especially in metabolism in Y. pestis, similar to previous findings in the acetylproteome of other bacteria (37, 38).

### Comparative acetylome analysis of *cobB* and *yfiQ* mutants versus WT Y. pestis identify YfiQ- or CobB-dependent acetylated proteins.

**FIG 3:**
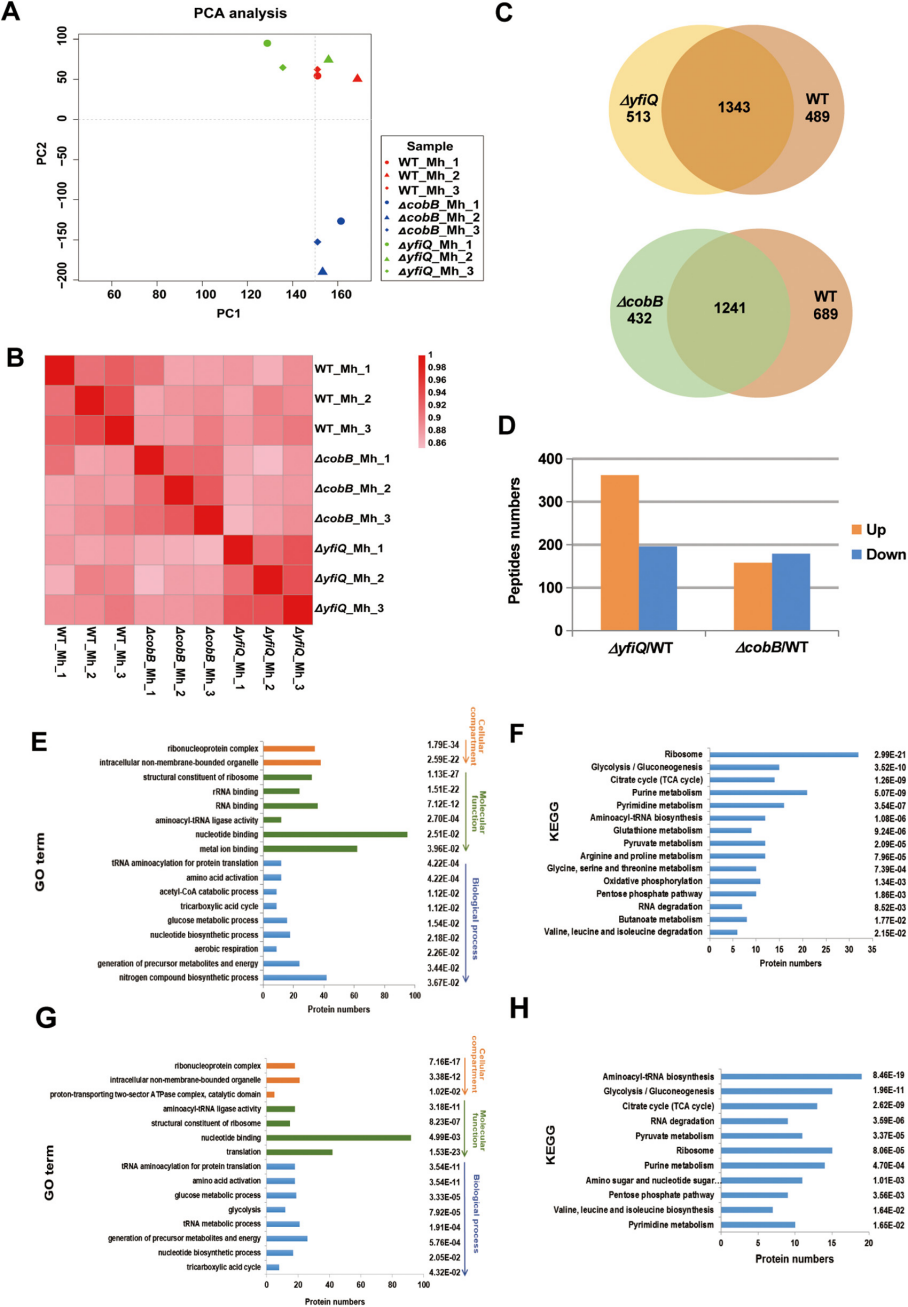
Comparative proteomics analysis between the *yfiQ* or *cobB* mutant and the WT Y. pestis. (A and B) Reproducibility of the quantification measurements of the WT strain and two mutants grown under the Mh condition between three replicates shown with a PCA scatterplot (A) and a heatmap of correlation coefficients (B). (C) Venn diagram of acetylated peptides detected in the *yfiQ* or *cobB* mutant and WT strain. (D) Numbers of acetylated peptides that were significantly differentially regulated in the two mutants compared to those in the WT strain. (E and F) Significantly upregulated proteins (*P* < 0.05) in the *yfiQ* mutant were classified according to GO terms and KEGG pathways. (G and H) Significantly downregulated proteins (*P* < 0.05) in the *cobB* mutant were classified according to GO terms (G) and KEGG pathways (H).

To gain functional insight into the protein acetylation modification mediated by YfiQ and CobB (the acetyltransferase and deacetylase, respectively) in Y. pestis, we compared the protein acetylation profiles of the *yfiQ* and *cobB* mutants to that of the WT bacteria. A PCA and heatmap of correlation coefficients were generated using R software from the data of the three replicates (Fig. 3A and B). A total of 2,345 acetylated peptides were detected in the *yfiQ* mutant and the WT strain; 513 of them were present only in the *yfiQ* mutant, and 489 were present only in WT, while 1,343 acetylated peptides could be quantitatively measured in both strains (Fig. 3C).

In the *yfiQ* mutant, 362 upregulated and 196 downregulated acetylated peptides were present relative to the WT strain (Fig. 3D). Of the 2,362 acetylated peptides detected in the *cobB* mutant and the WT strain, 432 peptides were only in the *cobB* mutant, 689 were only in the WT strain, and 1,241 peptides could be quantitatively measured in both strains (Fig. 3C). The abundance of 158 proteins increased and the abundance of 179 proteins decreased in the *cobB* mutant compared with the WT strain (Fig. 3D).

GO annotations and KEGG pathway enrichment analyses were performed for the acetylated proteins with at least one significantly altered site in the two mutants. Molecular functions associated with nucleotide binding and metal ion binding were significantly altered (Fig. 3E), and the KEGG pathways of ribosome and purine metabolism were significantly enriched when the *yfiQ* gene was deleted (Fig. 3F). Similar to previous reports, some of the acetylated proteins regulated by the *yfiQ* gene are known nucleotide-binding proteins, including elongation factor Tu (*tuf*) (39), DNA-directed RNA polymerase β-chain (*rpoC*) (40), and threonyl-tRNA synthetase (*thrS*) (41). Differentially regulated acetylated proteins in the *cobB* mutant were also significantly enriched in the GO term nucleotide binding and translation (Fig. 3G) and the KEGG pathways aminoacyl-tRNA biosynthesis and ribosome (Fig. 3H). Proteins with nucleotide binding and translation activity included the 50S ribosomal protein L7/L12 (*rplL*), the 16S rRNA processing protein (*rimM*), RNase E (*rne*), RNase T (*rnt*), the MarR-family transcriptional regulatory protein (*slyA*), cold shock protein (*cspC2*), heat shock protein GrpE (*grpE*), and amino acid decarboxylase (*adiA*). Taken together, our results show that YfiQ/CobB-mediated protein acetylation modifications have a broad regulatory effect on the physiological characteristics of Y. pestis.

In theory, knockout of *yfiQ* or *cobB* will result in a significant reduction or increase, respectively, of protein acetylation in Y. pestis. Therefore, we defined the criteria for significant changes in acetylated peptides between Δ*yfiQ* and the WT strain as follows: acetylated peptides with a log_2_ (Δ*yfiQ*/WT) value of less than 0 in each of the three replicates and a mean value of less than −1 were considered to be acetylated by YfiQ. Similarly, acetylated peptides with a log_2_ (Δ*cobB*/WT) value of greater than 0 in each test and a mean value of greater than 1 were considered to be modified by CobB. According to the above criteria, 24 acetylated peptides matching 23 acetylated proteins were acetylated by YfiQ (Table S2), and 470 acetylated peptides matching 315 acetylated proteins were deacetylated by CobB (Table S3). Far fewer YfiQ-dependent acetylated proteins were observed than CobB-dependent acetylated proteins, probably due to the presence of multiple acetyltransferases in the bacteria; however, CobB is the only deacetylase with confirmed functional activity in Y. pestis so far, although several bacterial deacetylases have been reported, such as YcgC, CddA, etc. (42–44). In addition, proteins can also be acetylated by the nonenzymatic acetyl-phosphate acetylation pathway (45).

The acetylated proteins modified by YfiQ are mostly related to metabolism and involve transcriptional regulation (CyaA and RpoC), translational regulation (TdcF3, a translational inhibitor protein), and other processes (Table S2). The acetylation modification of the cold shock protein (CspC2 and CspC3) and stress response regulatory protein (MsgA) by YfiQ probably represents a molecular mechanism affecting the stress response capability of Y. pestis. The 315 proteins deacetylated by CobB are mainly involved in metabolism and the cell cycle, followed by ribosomal proteins involved in cell composition (e.g., RplL, RplK, RplJ, and RpsA), stress response, and regulatory proteins. Many stress response proteins, such as cold shock proteins (CspE, CspC3, and CspC2), heat shock proteins (GrpE and HtpG), regulatory proteins of acid stress (AdiA), binary regulatory protein (PhoR and CpxR2), regulator protein (RcsB and SlyA), and virulence-related protein YMT, were modified by CobB (Table S3). In our previous study, virulence and stress response in the *cobB* mutant were significantly reduced (19). We hypothesize that the lack of deacetylase activity of CobB destroys the acetylation-deacetylation balance of the above proteins, resulting in an inability to respond to stressful stimuli through protein deacetylation regulation. In summary, the comparative acetylation proteomics of Y. pestis revealed potential acetylation proteins modified by YfiQ and CobB, which will help to identify their molecular targets.

### Acetylation of SlyA Lys73 affects its DNA binding activity.

**FIG 4:**
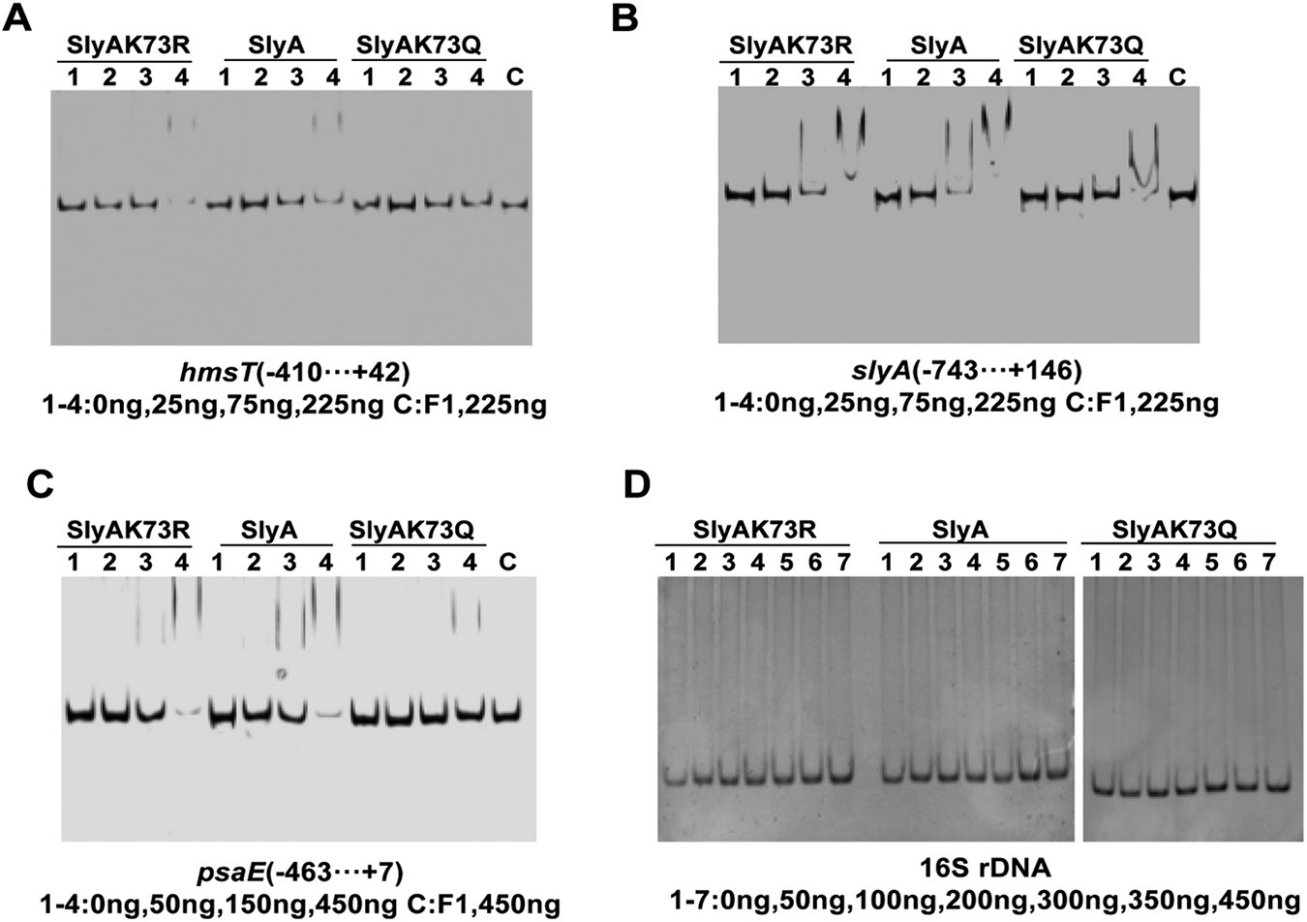
EMSAs of the binding affinity of SlyA and SlyA mutants to the promoter sequence of target genes. (A to C) The PCR-generated target DNA fragments of *hmsT* (A), *slyA* (B), and *psaE* (C) were incubated with increasing amounts of purified SlyA protein and two mutant proteins or an unrelated protein (purified F1 antigen). The reaction products were directly subjected to 6% (wt/vol) native PAGE. (D) An EMSA was conducted with a coding region of the 16S rRNA gene as a negative control.

Based on the acetylome analysis, we found that SlyA is acetylated at Lys73, and its deacetylation is probably modified by the deacetylase CobB (Table S3). To explore the effect of acetylation on protein function in Y. pestis, we used SlyA as the target to do further investigation. SlyA is a regulator belonging to the MarR/SlyA family that can regulate the expression of a large number of genes associated with virulence and biofilm formation, such as SPI-2-associated genes in Salmonella (46) and *pagP* in Escherichia coli (47). SlyA can directly bind to the promoter regions of *psaABC*, *psaEF*, and *hmsT* (48) as well as to itself to activate transcription (26, 49). We first constructed plasmids expressing the two Lys73 mutants of SlyA to simulate the permanent acetylated state (SlyA_K73Q_) and deacetylated state (SlyA_K73R_) of SlyA. Then, SlyA and its two mutants were expressed and purified (Fig. S3), and an electrophoretic mobility shift assay (EMSA) was performed to detect the binding ability of these proteins to the promoter sequences of the known target genes, including *hmsT* (Fig. 4A), *slyA* (Fig. 4B), and *psaE* (Fig. 4C). Both SlyA and SlyA_K73R_ bound to the promoter-proximal DNA fragment of each target gene in a dose-dependent manner *in vitro*, indicating that the two proteins have similar binding activity to each probe. However, DNA retardation was observed for the *slyA* promoter sequence only at higher concentrations of SlyA_K73Q_, while no band shift was observed for the other two probes. None of the three target genes could bind to the negative-control protein of F1 antigen, suggesting that the binding between the protein and the DNA probes was specific. This experiment also included a 269-bp DNA fragment in the coding region of the 16S rRNA gene as a negative-control probe; neither SlyA nor the point-mutant proteins at the tested concentrations could bind to the negative-control probe (Fig. 4D). In summary, the binding ability of the SlyA_K73R_ protein, which mimics the deacetylated state of SlyA, to the probes was similar to that of the SlyA protein, whereas the SlyA_K73Q_ protein, which mimics the acetylated state, had a decreased binding affinity. Our data reveal that the acetylation of Lys73 in the SlyA protein inhibits its binding to the promoter region of target genes.

### Biofilm formation ability is increased due to the K73Q mutation of SlyA.

**FIG 5:**
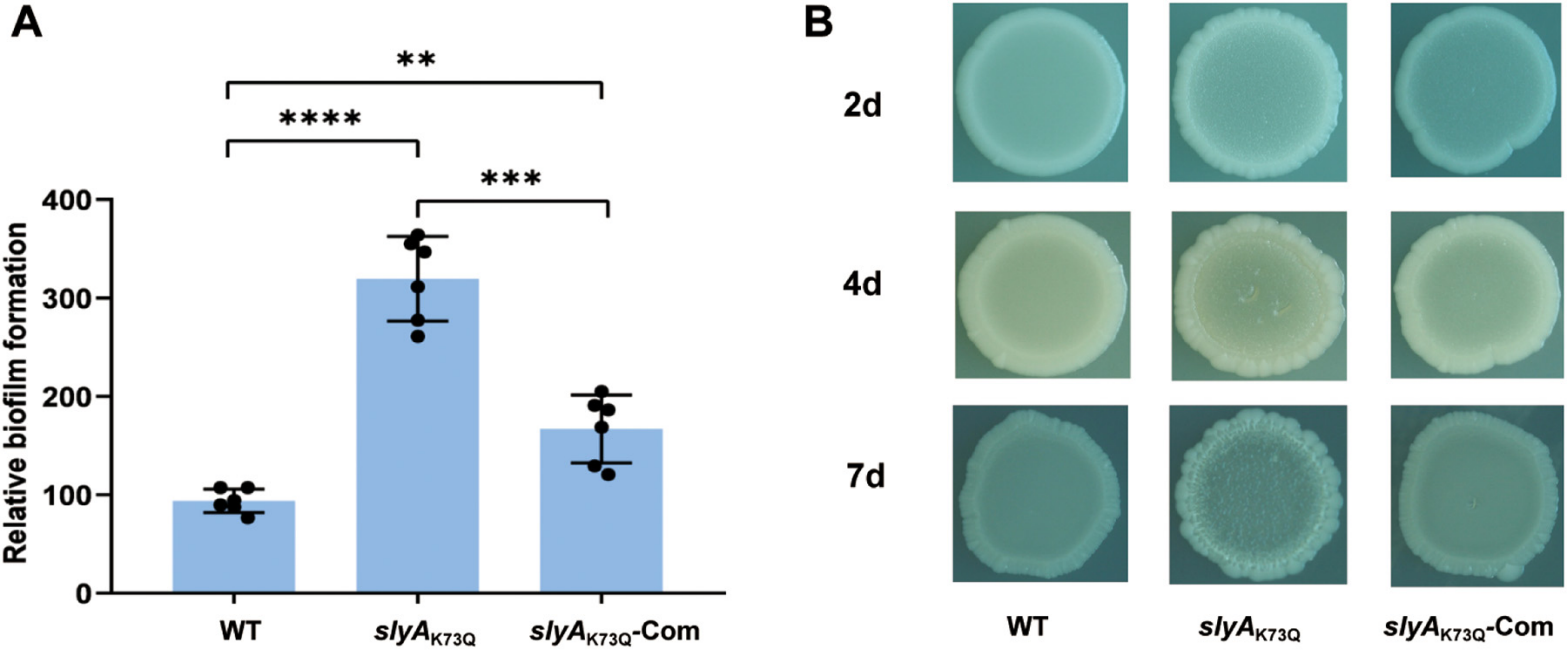
The *slyA*_K73Q_ mutant has an increased ability to form biofilms. (A) Crystal violet staining of *in vitro* biofilm masses. Y. pestis strains were grown in 24-well polystyrene plates, and the bacterial biomass adhering to the well walls was stained with crystal violet to determine the OD_570_ values. Planktonic cells were used to determine the OD_620_ values. The relative capacity to form biofilms for each strain tested is shown as the OD_570_/OD_620_ × 100 value. A one-way ANOVA with Fisher’s LSD test was used to analyze the differences between the various strains; **, *P* < 0.01; ***, *P* < 0.001; ****, *P* < 0.0001. (B) Bacterial rugose colony morphology assay. Glycerol stocks of Y. pestis strains were spotted on LB plates, followed by incubation for 1 week.

EMSA results indicated that acetylation of Lys73 in the SlyA protein inhibits its binding to the promoter region of target genes. To investigate the role of the K73Q mutation in SlyA in the virulence of strain 201, a 50% lethal dose (LD_50_) analysis was conducted with the *slyA*_K73Q_ isogenic mutant in BALB/c mice (Fig. S4 and Table S4). No significant virulence attenuation of the *slyA*_K73Q_ mutant was observed in mice infected with Y. pestis either intravenously or subcutaneously. The ability of Y. pestis to synthesize and form biofilms in the flea gut is important for flea-borne transmission of this pathogen (50, 51). HmsT and HmsD are diguanylate cyclases that synthesize c-di-GMP, which is required for biofilm formation (52, 53). EMSA results indicated that SlyA_K73Q_ has a significantly lower binding affinity to *hmsT*. Thus, we further sought to determine whether biofilm formation by Y. pestis is affected in *slyA*_K73Q_. Bacterial strains were inoculated into 24-well plates, and biofilms adhering to the walls of the wells were detected by crystal violet staining. Interestingly, biofilm formation of the *slyA*_K73Q_ mutant increased significantly compared to that of the WT strain. Mutant strains complemented with the SlyA expression plasmid showed a reduced ability to form a biofilm compared to the *slyA*_K73Q_ mutant, although it remained significantly higher than that of the WT strain, probably due to the overexpression of SlyA from pACYC184-SlyA (Fig. 5A). We further analyzed the colony morphology of various strains grown on agar plates (54). Irregular edges for *slyA*_K73Q_-mutant colonies could be seen as early as the second day, while colonies of the other two strains remained smooth. On the seventh day, both the WT strain and recombinant (*slyA*_K73Q_-Com) colonies developed rugose morphology to some extent but much less than that observed in *slyA*_K73Q_ colonies. Taken together, the colony morphology of *slyA*_K73Q_ was more rugose than that of the WT strain and *slyA*_K73Q_-Com, suggesting a higher biofilm formation capability of *slyA*_K73Q_ (Fig. 5B). These results are in line with the previous finding that SlyA directly represses the transcription of *hmsT* (26) and that mutation of K73Q in SlyA could relieve the repression effects on *hmsT*, as shown by EMSA in our study. These results demonstrate that acetylation modification of SlyA significantly influences biofilm formation of Y. pestis.

## DISCUSSION

Our study provides valuable resources on the lysine acetylome of Y. pestis grown under two typical conditions. Interestingly, more acetylated proteins and a higher degree of acetylation were detected in bacteria cultured under the Mh condition than in bacteria cultured under the Fv condition, suggesting that protein acetylation could be involved in adaptation of Y. pestis to the harsh environment of the mammalian host. Over 17% (Fv condition) and 20% (Mh condition) of acetylated proteins are highly acetylated, with some containing more than 30 acetylated sites, such as GroEL and YMT. This phenomenon has been found in many other bacteria, including *S. thermophiles* (33), M. tuberculosis (14), and P. aeruginosa. Why those proteins undergo such a high degree of acetylation and the underlying physiological significance are worthy of further investigation.

The bias for the acetylation site motif differs significantly among microorganisms. For example, no significant overall sequence recognition motif was detected among the acetylation sites of Salmonella enterica (55) or Erwinia amylovora (56). E. coli exhibits preferences for a glutamic acid or aspartic acid residue at the −1 position and for a histidine or tyrosine residue at the +1 position (57); in Bacillus subtilis, a glutamic acid, aspartic acid, lysine, or proline residue is more likely at the +1 position (12). This implies that the preference of acetylated modified motifs differs among bacterial acetylomes, unlike phosphorylation sites (14).

Comparing the abundances of acetylated peptides in the *yfiQ* and *cobB* mutants to those in the WT strain, 23 and 315 acetylated proteins were found to be potentially modified by YfiQ and CobB, respectively. This is probably due to the presence of multiple acetyltransferases as well as nonenzymatic acetyl-phosphate acetylation in the bacteria, whereas CobB is the only known deacetylase in *Y. pestis* so far.

We further demonstrate that acetylation of Lys73 inhibited the binding of SlyA to the promoter region of the target genes, resulting in significantly enhanced biofilm formation of Y. pestis. However, unlike previous findings that acetylation of Lys201 of PhoP (58) and Lys297 of Hild (59) impairs Salmonella virulence, the K73Q mutation in SlyA does not significantly affect the virulence of Y. pestis, although the possibility cannot be excluded that acetylation of SlyA on residues other than Lys73 affects virulence. More importantly, if protein acetylation substantially influences the activity of transcriptional regulators such as SlyA and PhoP, then bacteria can use lysine acetylation to fine-tune gene expression and improve its adaptation. The ubiquitous existence of protein acetylation and significant alterations between Y. pestis grown under Mh and Fv conditions highlight the importance of this PTM for the prompt response to external adverse environments.

## MATERIALS AND METHODS

### Bacterial strains and culture conditions.

We used Y. pestis biovar Microtus strain 201, which is avirulent to humans but highly virulent to mice (60). Experiments involving Y. pestis bacteria were performed in a biosafety level 2 laboratory. We constructed *cobB* and *yfiQ* mutants of Y. pestis strain 201 using the λ-red-based homologous recombination system, and the pKD46 helper plasmid was eliminated at 42°C (19). The wild-type (WT) strain and two mutant strains were grown in chemically defined TMH medium (61) with 2.5 mM Ca^2+^ at 26°C (mimicking the Fv condition) or without 2.5 mM Ca^2+^ at 37°C (mimicking the Mh condition), with shaking at 220 rpm. Escherichia coli BL21(DE3) cells were cultured in LB broth at 37°C with shaking at 220 rpm to express the recombinant proteins.

### Reagents and solutions.

The bicinchoninic acid (BCA) protein assay kit was from Thermo (Waltham, MA, USA). The protease inhibitor cocktail was from Roche (Basel, Switzerland). Tricustin A (TSA), niacinamide (NAM), and sodium butyrate were from Sigma-Aldrich (St. Louis, MO, USA). The acetylated peptide enrichment kit was from Cell Signaling Technology (Danforth, MA, USA). Sep-pak C_18_ packings were from Waters (MA, USA). The Fast mutagenesis system was from TransGen Biotech (Beijing, China). Nickel-nitrilotriacetic acid (NTA) agarose and QIAquick PCR purification kits were from Qiagen (Dusseldorf, North Rhine-Westphalia, Germany). Restriction endonucleases were from New England BioLabs (Ipswich, MA, USA). Goldview nucleic acid gel stain was from Biomed (Beijing, China).

### Extraction of bacterial proteins.

WT Y. pestis and the two mutant strains were cultured in TMH with 2.5 mM Ca^2+^ at 26°C with shaking at 220 rpm to the log phase, and each strain was subcultured at a ratio of 1:20 into 200 mL of fresh TMH medium and incubated at 26°C. After reaching an optical density at 600 nm (OD_600_) of ≈1.0, bacterial cultures were divided equally into two tubes and centrifuged at 1,700 × *g* for 10 min. After removing the supernatants, one-half of the bacterial cell pellets was resuspended in 100 mL of fresh Ca^2+^-free TMH and cultured at 37°C at 220 rpm; the other half was resuspended in 100 mL of fresh TMH with 2.5 mM Ca^2+^ and cultured at 26°C at 220 rpm.

Bacteria were harvested at the exponential growth phase by centrifugation. Bacterial pellets were washed twice with phosphate-buffered saline (PBS) and resuspended in 30 mL of chilled lysis buffer {7 M urea, 2 M thiourea, 4% 3-[(3-cholamidopropyl)-dimethylammonio]-1-propanesulfonate [CHAPS], and 50 mM Tris-HCl, pH 8.5}, followed by sonication in an ice-water bath. Cell lysates were centrifuged at 10,000 × *g* for 30 min at 4°C to collect the supernatant. Then, 1 mM dithiothreitol (DTT) was added, and the mixture was incubated for 1 h. Iodoacetamide was added to block the sulfhydryl for 1 h, and samples were kept in the dark. Five times the volume of acetone was added, and proteins were precipitated overnight at −20°C. The next morning, proteins were separated by centrifugation, washed with ice-cold acetone, and air dried. Protein pellets were redissolved with redissolve buffer (8 M urea and 10 mM HEPES, pH 8.0), and the insoluble proteins were discarded.

### Protein sample pretreatment.

Protein concentrations were measured with a BCA protein assay kit. Proteins (20 mg) in 8 M urea were diluted with 50 mM ammonium bicarbonate to reduce the concentration of urea to less than 1.5 M. The lysate was digested with trypsin (1/50 the mass of the protein) at 37°C for 16 h. Tryptic digestion was quenched by the addition of 0.1% trifluoroacetic acid (TFA). The solution was clarified by centrifugation at 2,000 × *g* for 10 min at room temperature. The resulting peptides were then fractionated and desalted using reversed-phase C_18_ columns self-packed with C_18_ material (40 μm, 60-Å pore size; Agilent Technologies, Santa Clara, CA). The eluent of peptides was collected, dried in a SpeedVac, and stored at −20°C until further analysis.

### Enrichment of acetylated peptides.

Agarose beads coated with anti-acetyl-lysine antibody were washed three times with PBS before use. Tryptic peptides were redissolved in immunoaffinity purification (IAP) buffer, and the insoluble particles were removed by centrifugation. The prewashed agarose-conjugated anti-acetyl-lysine antibody was added to the peptide solution and incubated at 4°C for 2 h with rotary shaking. Beads were washed three times with 1 mL of IAP buffer and then three times with 1 mL of ice-cold water. The bonded peptides were eluted with 0.15% TFA in water. Eluted peptides were desalted using STAGE tips and then concentrated for mass spectrometry (MS) analysis.

### Liquid chromatography-tandem mass spectrometry analysis.

Peptides were separated on a Dionex nano-high-performance liquid chromatography (HPLC)/mass spectrometry system (Dionex, Sunnyvale, CA) and analyzed online using an electrospray ion-trap mass spectrometer (HCTultra; Bruker Daltonics, Bremen, Germany). For each analysis, the sample was trapped on a C_18_ precolumn (20-mm length × 100-μm inner diameter) containing C_18_ resin (5-μm particle size and 100-Å pore diameter; Dionex) and then on a C_18_ reversed-phase analytical column (150-mm length × 75-μm inner diameter) containing C_18_ resin (2-μm particle size and 100-Å pore diameter; Dionex).

Desalination was on a precolumn (20-mm length × 100-μm inner diameter) containing C_18_ resin, and peptides were separated with an analytical column (150-mm length × 75-μm inner diameter) containing C_18_ resin (2-μm particle size and 100-Å pore diameter). The peptides were eluted from the column with a linear solvent gradient (A: 0.1% formic acid [FA] in water; B: 100% acetonitrile/0.1% FA) for 120 min using an HPLC gradient from 5% to 90% HPLC buffer B at a flow rate of 0.25 μL/min.

The mass spectrometry parameters were set as follows. An Xcalibur 2.1.3 was used to perform full scanning in Orbitrap with a resolution of 70,000, a scanning range of 300 to 1,800 *m*/*z*, and an automatic gain control (AGC) of 3 × 10^6^. The second-level scan adopted the data-dependent acquisition method with a resolution of 30,000, an AGC of 5 × 10^4^, and a maximum injection time (IT) of 120 ms. After each level, a maximum of 20 second-level scans was performed. The isolation window of the second-level scans was 1.2 Da, dynamic exclusion time was 60 s, and normalized collisional energy (NCE) was 30; ions that were more than 10 valences did not have a second-level scan.

### Protein identification and quantification.

The mass spectrometric data sets were searched against the Y. pestis 91001 database (downloaded from NCBI, GenBank assembly accession number GCA_000007885.1) using the SEQUEST search engine of the Proteome Discoverer (PD) software package (version 2.1, Thermo Scientific) and MaxQuant software with an overall false-discovery rate (FDR) for peptides of less than 1%. The search criteria were as follows: full tryptic specificity was required, up to four missed cleavage sites per peptide were allowed, carbamidomethylation of cysteine was set as a fixed modification and oxidation of methionine and acetylation of lysine were set as variable modifications, precursor ion mass tolerances were set at 10 ppm for all mass spectra acquired in an orbitrap mass analyzer, and the fragment ion mass tolerance was set at 20 millimass unit (mmu) for all MS2 spectra acquired.

### Functional annotation and analysis.

Functional categories were assigned to each protein according to the genome annotation of Y. pestis CO92. Web Logo (https://weblogo.berkeley.edu/logo.cgi) was used to analyze the 30 amino acids flanking the acetylated sites to explore whether there was a conserved acetylation motif in Y. pestis. GO term enrichment analysis in biological process, cellular compartment, and molecular function was conducted using web-based PANTHER bioinformatics tools (http://www.pantherdb.org/) (36). KEGG pathway enrichment was performed using DAVID bioinformatics tools (https://david.ncifcrf.gov/) (62). Significance of the enrichment was tested with a Fisher exact test. A *P* value of <0.05 and an FDR of <0.05 were considered significant. Acetylated peptides with a |log_2_ (mutant/WT, intensity)| of ≥1 were interpreted as differentially expressed peptides (DEPs) in comparative acetylome analysis. The reproducibility of the quantification measurements between three replicates was analyzed with R software (version 4.2.1, https://www.R-project.org/). Batch effects removal was performed with the R package ConQuR (32). ConQuR is a comprehensive method that shows advantages in removing batch effects while preserving the signals of interest. A PCA was performed with the R function prcomp (29), and scatterplots were painted with the R package ggplot2 (30). Heatmaps were generated using the R package pheatmap (31).

### DNA manipulations.

The *slyA* coding sequence was amplified and cloned into pET28a (Novagen). Two point-mutation plasmids pET28a-*slyA* (K73R) and pET28a-*slyA* (K73Q) were amplified using appropriate primers (SlyA[K73R]-F/R and SlyA[K73Q]-F/R) (Table S5 in the supplemental material), and pET28a-*slyA* was used as a template with a point mutation construction kit (Fast Mutagenesis System, TransGen). Positive clones were identified by PCR using a SlyA-EXP-F/R primer pair (Table S5) sequenced to ensure the correct sequence of the constructed plasmid. Then, the successfully constructed plasmids were chemically transformed into BL21(DE3) cells to obtain strains expressing SlyA_K73R_ or SlyA_K73Q_. The upstream and downstream homology arms of *slyA* were amplified from strain 201 using the primer pairs pDS-*slyA*-F/R and were cloned into the suicide plasmid pDS132, and the *slyA*_K73Q_ isogenic mutant strain of Y. pestis 201 was constructed as previously described (63, 64). For transcomplementation, a DNA fragment containing 500 bp of upstream sequence and the full *slyA* coding sequence was amplified using the primer pairs *slyA*-com-F/R and then cloned into pACYC184. The recombinant plasmids pACYC184-*slyA* were electroporated into the *slyA*_K73Q_ mutant strain to generate the s*lyA*_K73Q_-Com strain. The primer sequences used are shown in Table S5.

### Protein expression and purification.

E. coli BL21(DE3) cells expressing SlyA or its variants were cultured in LB medium at 37°C with shaking at 220 rpm. For expression measures of SlyA and the two mutants, the overnight culture from a single colony was used to inoculate 200 mL of LB medium. Cells were grown with vigorous shaking at 37°C to an OD_620_ of 0.8 and induced with 0.1 mM isopropyl-β-d-thiogalactoside (IPTG) at 26°C for 12 h. Bacterial cells were harvested, resuspended in PBS, and lysed by sonication. Bacterial lysates were centrifuged at 12,000 × *g* for 20 min to remove bacterial debris, and the soluble recombinant proteins were purified by affinity chromatography using NTA agarose. The protein purity was verified by 10% SDS-PAGE. All steps after cell harvest were performed at 4°C, and purified proteins were stored at −80°C.

### Electrophoretic mobility shift assay (EMSA).

Primers were designed according to the promoter region of the target genes (Table S5), and the entire upstream promoter-proximal DNA region of each target gene was amplified by PCR. The amplification products were concentrated at approximately 100 ng/μL and stored at −20°C for later use. A BCA protein assay kit was used to determine the concentrations of SlyA, SlyA_K73R_, and SlyA_K73Q_ extracted above to ensure equal loading of samples. DNA binding was performed in a 10-μL reaction volume containing 2 μL of 5× binding buffer, increasing amounts of SlyA or the two mutant proteins, and water to achieve a total volume of 9 μL. Target promoter-proximal DNA (1 μL at 100 ng/μL) was added to each reaction tube followed by another incubation for 20 min at room temperature. Two controls were included in each EMSA: (i) a negative probe as a nonspecific DNA competitor (the coding region of the 16S rRNA gene) and (ii) a nonspecific protein competitor (purified F1 antigen, the protective antigen from Y. pestis) (65). Products were loaded onto a 6% (wt/vol) native polyacrylamide gel and electrophoresed in 1.0× Tris-borate (TB) buffer at 150 V and low temperature. After staining with GoldView dye, the gel was imaged with a UV transilluminator.

### Crystal violet staining of *in vitro* biofilm masses.

Bacterial biofilm formation ability was analyzed using the crystal violet staining method as described previously (19). The WT strain, *slyA*_K73Q_ mutant strain, and *slyA*_K73Q_-Com strain were cultured in LB culture medium at 26°C with shaking at 220 rpm to the log phase. Each strain was subcultured at a ratio of 1:20 into 24-well tissue culture plates with 1 mL of fresh LB in each well and then incubated at 26°C for 24 h. The culture medium containing the planktonic cells was removed from each well to determine the OD_620_. Wells with adherent biofilms were washed gently three times with 2 mL of water and then fixed at 80°C for 15 min. Surface-attached cells were stained with 3 mL of 0.1% crystal violet for 15 min. The solution was discarded, wells were washed three times with 3 mL of water, and the pigment in the wells was dissolved with 3 mL of dimethyl sulfoxide. The OD_570_ values were recorded, and the OD_570_/OD_620_ × 100 was calculated. One-way analysis of variance (ANOVA) with Fisher’s least significant difference (LSD) test was performed using GraphPad Prism 8 software to analyze differences between the various strains.

### Colony morphology assay.

Aliquots of the glycerol stock of the three Y. pestis strains were spotted on LB agar plates and incubated for 1 week at 26°C (66). Images of colony morphologies were acquired using a Canon 60D DSLR camera (Canon, Inc., Tokyo, Japan).

### Determination of LD_50_.

The Y. pestis strain 201 used in this study is avirulent to humans but highly virulent to mice, and animal biosafety level 3 facilities were used for infected animal experiments at Beijing Institute of Epidemiology and Microbiology. The *slyA*_K73Q_ mutant of the 201 strain was cultured overnight in LB at 26°C. The bacterial culture was serially diluted 10-fold in PBS to bacterial suspensions of 1 to 10^4^ CFU/mL. Actual numbers of CFU/mL were determined by plating the dilutions on agar plates. BALB/c mice were purchased from the Beijing Vital River Laboratory Animal Technology Company. All animal experiments were carried out in strict accordance to the Guidelines for the Welfare and Ethics of Laboratory Animals of China and were approved by the Institutional Animal Care Committee of Beijing Institute of Epidemiology and Microbiology (protocol number IACUCDWZX-2020-016). For each of the test strains, five groups of 6- to 8-week-old female BALB/c mice (*n* = 6 per group) were challenged by subcutaneous injection at inguinal or intravenous injection via the vena caudalis with serial 10-fold dilutions of bacterial cell suspension. Mice were observed daily for 14 days, and the LD_50_ was calculated using the Reed-Muench method (67).

### Data availability.

All the mass spectrometry proteomics data discussed in the manuscript have been deposited to the ProteomeXchange Consortium (https://proteomecentral.proteomexchange.org) via the iProX partner repository with the data set identifier PXD038124.

## References

[1] Bali G, Foston MB, O’Neill HM, Evans BR, He J, Ragauskas AJ. 2013. The effect of deuteration on the structure of bacterial cellulose. Carbohydr Res 374:82–88. doi:10.1016/j.carres.2013.04.009. 23651632

[2] Keller M, Spyrou MA, Scheib CL, Neumann GU, Kröpelin A, Haas-Gebhard B, Päffgen B, Haberstroh J, Ribera I Lacomba A, Raynaud C, Cessford C, Durand R, Stadler P, Nägele K, Bates JS, Trautmann B, Inskip SA, Peters J, Robb JE, Kivisild T, Castex D, McCormick M, Bos KI, Harbeck M, Herbig A, Krause J. 2019. Ancient *Yersinia pestis* genomes from across Western Europe reveal early diversification during the First Pandemic (541–750). Proc Natl Acad Sci USA 116:12363–12372. doi:10.1073/pnas.1820447116. 31164419 PMC6589673

[3] Andrianaivoarimanana V, Piola P, Wagner DM, Rakotomanana F, Maheriniaina V, Andrianalimanana S, Chanteau S, Rahalison L, Ratsitorahina M, Rajerison M. 2019. Trends of human plague, Madagascar, 1998–2016. Emerg Infect Dis 25:220–228. doi:10.3201/eid2502.171974. 30666930 PMC6346457

[4] Branda JA, Ruoff K. 2002. Bioterrorism. Clinical recognition and primary management. Am J Clin Pathol 117 Suppl:S116–S123. doi:10.1309/5G7E-F5HQ-3G6E-VQMB. 14569808

[5] Phillips DM. 1963. The presence of acetyl groups of histones. Biochem J 87:258–263. doi:10.1042/bj0870258. 13943142 PMC1201885

[6] Mischerikow N, Heck AJ. 2011. Targeted large-scale analysis of protein acetylation. Proteomics 11:571–589. doi:10.1002/pmic.201000397. 21246731

[7] Kim SC, Sprung R, Chen Y, Xu Y, Ball H, Pei J, Cheng T, Kho Y, Xiao H, Xiao L, Grishin NV, White M, Yang X-J, Zhao Y. 2006. Substrate and functional diversity of lysine acetylation revealed by a proteomics survey. Mol Cell 23:607–618. doi:10.1016/j.molcel.2006.06.026. 16916647

[8] Choudhary C, Kumar C, Gnad F, Nielsen ML, Rehman M, Walther TC, Olsen JV, Mann M. 2009. Lysine acetylation targets protein complexes and co-regulates major cellular functions. Science 325:834–840. doi:10.1126/science.1175371. 19608861

[9] Kim C, Hesek D, Zajicek J, Vakulenko SB, Mobashery S. 2006. Characterization of the bifunctional aminoglycoside-modifying enzyme ANT(3'')-Ii/AAC(6')-IId from *Serratia marcescens*. Biochemistry 45:8368–8377. doi:10.1021/bi060723g. 16819836

[10] Zhao S, Xu W, Jiang W, Yu W, Lin Y, Zhang T, Yao J, Zhou L, Zeng Y, Li H, Li Y, Shi J, An W, Hancock SM, He F, Qin L, Chin J, Yang P, Chen X, Lei Q, Xiong Y, Guan K-L. 2010. Regulation of cellular metabolism by protein lysine acetylation. Science 327:1000–1004. doi:10.1126/science.1179689. 20167786 PMC3232675

[11] Kouzarides T. 2000. Acetylation: a regulatory modification to rival phosphorylation? EMBO J 19:1176–1179. doi:10.1093/emboj/19.6.1176. 10716917 PMC305658

[12] Kim D, Yu BJ, Kim JA, Lee YJ, Choi SG, Kang S, Pan JG. 2013. The acetylproteome of Gram-positive model bacterium *Bacillus subtilis*. Proteomics 13:1726–1736. doi:10.1002/pmic.201200001. 23468065

[13] ATLAS Collaboration, Aad G, Abajyan T, Abbott B, Abdallah J, Abdel Khalek S, Abdelalim AA, Abdinov O, Aben R, Abi B, Abolins M, AbouZeid OS, Abramowicz H, Abreu H, Acharya BS, Adamczyk L, Adams DL, Addy TN, Adelman J, Adomeit S, Adragna P, Adye T, Aefsky S, Aguilar-Saavedra JA, Agustoni M, Aharrouche M, Ahlen SP, Ahles F, Ahmad A, Ahsan M, Aielli G, Åkesson TPA, Akimoto G, Akimov AV, Alam MA, Albert J, Albrand S, Aleksa M, Aleksandrov IN, Alessandria F, Alexa C, Alexander G, Alexandre G, Alexopoulos T, Alhroob M, Aliev M, Alimonti G, Alison J, Allbrooke BMM, Allison LJ, Allport PP, et al. 2013. Measurement of the [formula: see text] production cross section in the tau + jets channel using the ATLAS detector. Eur Phys J C Part Fields 73:2328. doi:10.1140/epjc/s10052-013-2328-7. 25814855 PMC4371093

[14] Liu F, Yang M, Wang X, Yang S, Gu J, Zhou J, Zhang XE, Deng J, Ge F. 2014. Acetylome analysis reveals diverse functions of lysine acetylation in *Mycobacterium tuberculosis*. Mol Cell Proteomics 13:3352–3366. doi:10.1074/mcp.M114.041962. 25180227 PMC4256489

[15] Sang Y, Ren J, Ni J, Tao J, Lu J, Yao YF. 2016. Protein acetylation is involved in *Salmonella enterica* serovar Typhimurium virulence. J Infect Dis 213:1836–1845. doi:10.1093/infdis/jiw028. 26810370

[16] Wei W, Liu X, Chen J, Gao S, Lu L, Zhang H, Ding G, Wang Z, Chen Z, Shi T, Li J, Yu J, Wong J. 2017. Class I histone deacetylases are major histone decrotonylases: evidence for critical and broad function of histone crotonylation in transcription. Cell Res 27:898–915. doi:10.1038/cr.2017.68. 28497810 PMC5518989

[17] Song Y, Tong Z, Wang J, Wang L, Guo Z, Han Y, Zhang J, Pei D, Zhou D, Qin H, Pang X, Han Y, Zhai J, Li M, Cui B, Qi Z, Jin L, Dai R, Chen F, Li S, Ye C, Du Z, Lin W, Wang J, Yu J, Yang H, Wang J, Huang P, Yang R. 2004. Complete genome sequence of *Yersinia pestis* strain 91001, an isolate avirulent to humans. DNA Res 11:179–197. doi:10.1093/dnares/11.3.179. 15368893

[18] Parkhill J, Wren BW, Thomson NR, Titball RW, Holden MT, Prentice MB, Sebaihia M, James KD, Churcher C, Mungall KL, Baker S, Basham D, Bentley SD, Brooks K, Cerdeño-Tárraga AM, Chillingworth T, Cronin A, Davies RM, Davis P, Dougan G, Feltwell T, Hamlin N, Holroyd S, Jagels K, Karlyshev AV, Leather S, Moule S, Oyston PC, Quail M, Rutherford K, Simmonds M, Skelton J, Stevens K, Whitehead S, Barrell BG. 2001. Genome sequence of *Yersinia pestis*, the causative agent of plague. Nature 413:523–527. doi:10.1038/35097083. 11586360

[19] Liu W, Tan Y, Cao S, Zhao H, Fang H, Yang X, Wang T, Zhou Y, Yan Y, Han Y, Song Y, Bi Y, Wang X, Yang R, Du Z. 2018. Protein acetylation mediated by YfiQ and CobB is involved in the virulence and stress response of *Yersinia pestis*. Infect Immun 86:e00224-18. doi:10.1128/IAI.00224-18. 29610260 PMC5964501

[20] Wolfe AJ. 2016. Bacterial protein acetylation: new discoveries unanswered questions. Curr Genet 62:335–341. doi:10.1007/s00294-015-0552-4. 26660885 PMC4826803

[21] Imai S, Guarente L. 2010. Ten years of NAD-dependent SIR2 family deacetylases: implications for metabolic diseases. Trends Pharmacol Sci 31:212–220. doi:10.1016/j.tips.2010.02.003. 20226541 PMC3526941

[22] Greiss S, Gartner A. 2009. Sirtuin/Sir2 phylogeny, evolutionary considerations and structural conservation. Mol Cells 28:407–415. doi:10.1007/s10059-009-0169-x. 19936627 PMC3710699

[23] Pujol C, Grabenstein JP, Perry RD, Bliska JB. 2005. Replication of *Yersinia pestis* in interferon gamma-activated macrophages requires *ripA*, a gene encoded in the pigmentation locus. Proc Natl Acad Sci USA 102:12909–12914. doi:10.1073/pnas.0502849102. 16120681 PMC1200267

[24] Lukaszewski RA, Kenny DJ, Taylor R, Rees DG, Hartley MG, Oyston PC. 2005. Pathogenesis of *Yersinia pestis* infection in BALB/c mice: effects on host macrophages and neutrophils. Infect Immun 73:7142–7150. doi:10.1128/IAI.73.11.7142-7150.2005. 16239508 PMC1273833

[25] Deochand DK, Grove A. 2017. MarR family transcription factors: dynamic variations on a common scaffold. Crit Rev Biochem Mol Biol 52:595–613. doi:10.1080/10409238.2017.1344612. 28670937

[26] Liu L, Fang H, Yang H, Zhang Y, Han Y, Zhou D, Yang R. 2016. Reciprocal regulation of *Yersinia pestis* biofilm formation and virulence by RovM and RovA. Open Biol 6:150198. doi:10.1098/rsob.150198. 26984293 PMC4821237

[27] Cathelyn JS, Crosby SD, Lathem WW, Goldman WE, Miller VL. 2006. RovA, a global regulator of *Yersinia pestis*, specifically required for bubonic plague. Proc Natl Acad Sci USA 103:13514–13519. doi:10.1073/pnas.0603456103. 16938880 PMC1569194

[28] Wu HY, Huang FY, Chang YC, Hsieh MC, Liao PC. 2008. Strategy for determination of *in vitro* protein acetylation sites by using isotope-labeled acetyl coenzyme A and liquid chromatography-mass spectrometry. Anal Chem 80:6178–6189. doi:10.1021/ac800440r. 18616279

[29] Libório MP, da Silva Martinuci O, Machado AMC, Machado-Coelho TM, Laudares S, Bernardes P. 2022. Principal component analysis applied to multidimensional social indicators longitudinal studies: limitations and possibilities. GeoJournal 87:1453–1468.

[30] Cupp MA, Cariolou M, Tzoulaki I, Aune D, Evangelou E, Berlanga-Taylor AJ. 2020. Neutrophil to lymphocyte ratio and cancer prognosis: an umbrella review of systematic reviews and meta-analyses of observational studies. BMC medicine 18:1–16. doi:10.1186/s12916-020-01817-1. 33213430 PMC7678319

[31] Jansen J, Reimer KC, Nagai JS, Varghese FS, Overheul GJ, de Beer M, Roverts R, Daviran D, Fermin LA, Willemsen B. 2022. SARS-CoV-2 infects the human kidney and drives fibrosis in kidney organoids. Cell Stem Cell 29:217–231.e218. doi:10.1016/j.stem.2021.12.010. 35032430 PMC8709832

[32] Ling W, Lu J, Zhao N, Lulla A, Plantinga AM, Fu W, Zhang A, Liu H, Song H, Li Z, Chen J, Randolph TW, Koay WLA, White JR, Launer LJ, Fodor AA, Meyer KA, Wu MC. 2022. Batch effects removal for microbiome data via conditional quantile regression. Nat Commun 13:5418. doi:10.1038/s41467-022-33071-9. 36109499 PMC9477887

[33] Okanishi H, Kim K, Masui R, Kuramitsu S. 2013. Acetylome with structural mapping reveals the significance of lysine acetylation in *Thermus thermophilus*. J Proteome Res 12:3952–3968. doi:10.1021/pr400245k. 23901841

[34] Ouidir T, Cosette P, Jouenne T, Hardouin J. 2015. Proteomic profiling of lysine acetylation in *Pseudomonas aeruginosa* reveals the diversity of acetylated proteins. Proteomics 15:2152–2157. doi:10.1002/pmic.201500056. 25900529

[35] Ouidir T, Kentache T, Hardouin J. 2016. Protein lysine acetylation in bacteria: current state of the art. Proteomics 16:301–309. doi:10.1002/pmic.201500258. 26390373

[36] Mi H, Ebert D, Muruganujan A, Mills C, Albou LP, Mushayamaha T, Thomas PD. 2021. PANTHER version 16: a revised family classification, tree-based classification tool, enhancer regions and extensive API. Nucleic Acids Res 49:D394–D403. doi:10.1093/nar/gkaa1106. 33290554 PMC7778891

[37] Li D, Lv B, Tan L, Yang Q, Liang W. 2016. Acetylome analysis reveals the involvement of lysine acetylation in diverse biological processes in *Phytophthora sojae*. Sci Rep 6:29897. doi:10.1038/srep29897. 27412925 PMC4944153

[38] Zhou X, Qian G, Yi X, Li X, Liu W. 2016. Systematic analysis of the lysine acetylome in *Candida albicans*. J Proteome Res 15:2525–2536. doi:10.1021/acs.jproteome.6b00052. 27297460

[39] Koenigs A, Zipfel PF, Kraiczy P. 2015. Translation elongation factor Tuf of *Acinetobacter baumannii* is a plasminogen-binding protein. PLoS One 10:e0134418. doi:10.1371/journal.pone.0134418. 26230848 PMC4521846

[40] Guo Y, Winkler J, Kao KC. 2017. Insights on osmotic tolerance mechanisms in *Escherichia coli* gained from an *rpoC* mutation. Bioengineering (Basel) 4:61. doi:10.3390/bioengineering4030061. 28952540 PMC5615307

[41] Luo D, Condon C, Grunberg-Manago M, Putzer H. 1998. *In vitro* and *in vivo* secondary structure probing of the *thrS* leader in *Bacillus subtilis*. Nucleic Acids Res 26:5379–5387. doi:10.1093/nar/26.23.5379. 9826762 PMC148014

[42] Castano-Cerezo S, Bernal V, Post H, Fuhrer T, Cappadona S, Sanchez-Diaz NC, Sauer U, Heck AJ, Altelaar AF, Canovas M. 2014. Protein acetylation affects acetate metabolism, motility and acid stress response in *Escherichia coli*. Mol Syst Biol 10:762. doi:10.15252/msb.20145227. 25518064 PMC4299603

[43] Liu X, Yang M, Liu Y, Ge F, Zhao J. 2020. Structural and functional insights into a lysine deacylase in the cyanobacterium *Synechococcus* sp. PCC 7002. Plant Physiol 184:762–776. doi:10.1104/pp.20.00583. 32719110 PMC7536712

[44] Tu S, Guo S-J, Chen C-S, Liu C-X, Jiang H-W, Ge F, Deng J-Y, Zhou Y-M, Czajkowsky DM, Li Y, Qi B-R, Ahn Y-H, Cole PA, Zhu H, Tao S-C. 2015. YcgC represents a new protein deacetylase family in prokaryotes. eLife 4:e05322. doi:10.7554/eLife.05322. 26716769 PMC4709262

[45] VanDrisse CM, Escalante-Semerena JC. 2019. Protein acetylation in bacteria. Annu Rev Microbiol 73:111–132. doi:10.1146/annurev-micro-020518-115526. 31091420 PMC6736716

[46] Linehan SA, Rytkonen A, Yu XJ, Liu M, Holden DW. 2005. SlyA regulates function of *Salmonella* pathogenicity island 2 (SPI-2) and expression of SPI-2-associated genes. Infect Immun 73:4354–4362. doi:10.1128/IAI.73.7.4354-4362.2005. 15972530 PMC1168564

[47] Chalabaev S, Chauhan A, Novikov A, Iyer P, Szczesny M, Beloin C, Caroff M, Ghigo JM. 2014. Biofilms formed by Gram-negative bacteria undergo increased lipid a palmitoylation, enhancing *in vivo* survival. mBio 5:e01116-14. doi:10.1128/mBio.01116-14. 25139899 PMC4147861

[48] Zhang Y, Wang L, Fang N, Qu S, Tan Y, Guo Z, Qiu J, Zhou D, Yang R. 2013. Reciprocal regulation of pH 6 antigen gene loci by PhoP and RovA in *Yersinia pestis* biovar Microtus. Future Microbiol 8:271–280. doi:10.2217/fmb.12.146. 23374131

[49] Heroven AK, Nagel G, Tran HJ, Parr S, Dersch P. 2004. RovA is autoregulated and antagonizes H-NS-mediated silencing of invasin and *rovA* expression in *Yersinia pseudotuberculosis*. Mol Microbiol 53:871–888. doi:10.1111/j.1365-2958.2004.04162.x. 15255899

[50] Hinnebusch BJ, Erickson DL. 2008. *Yersinia pestis* biofilm in the flea vector and its role in the transmission of plague. Curr Top Microbiol Immunol 322:229–248. doi:10.1007/978-3-540-75418-3_11. 18453279 PMC3727414

[51] Zhou D, Yang R. 2011. Formation and regulation of *Yersinia* biofilms. Protein Cell 2:173–179. doi:10.1007/s13238-011-1024-3. 21380640 PMC4875308

[52] Bobrov AG, Kirillina O, Ryjenkov DA, Waters CM, Price PA, Fetherston JD, Mack D, Goldman WE, Gomelsky M, Perry RD. 2011. Systematic analysis of cyclic di-GMP signalling enzymes and their role in biofilm formation and virulence in *Yersinia pestis*. Mol Microbiol 79:533–551. doi:10.1111/j.1365-2958.2010.07470.x. 21219468 PMC3058942

[53] Sun YC, Koumoutsi A, Jarrett C, Lawrence K, Gherardini FC, Darby C, Hinnebusch BJ. 2011. Differential control of *Yersinia pestis* biofilm formation *in vitro* and in the flea vector by two c-di-GMP diguanylate cyclases. PLoS One 6:e19267. doi:10.1371/journal.pone.0019267. 21559445 PMC3084805

[54] Sun YC, Hinnebusch BJ, Darby C. 2008. Experimental evidence for negative selection in the evolution of a *Yersinia pestis* pseudogene. Proc Natl Acad Sci USA 105:8097–8101. doi:10.1073/pnas.0803525105. 18523005 PMC2430365

[55] Wang Q, Zhang Y, Yang C, Xiong H, Lin Y, Yao J, Li H, Xie L, Zhao W, Yao Y, Ning Z-B, Zeng R, Xiong Y, Guan K-L, Zhao S, Zhao G-P. 2010. Acetylation of metabolic enzymes coordinates carbon source utilization and metabolic flux. Science 327:1004–1007. doi:10.1126/science.1179687. 20167787 PMC4183141

[56] Wu X, Vellaichamy A, Wang D, Zamdborg L, Kelleher NL, Huber SC, Zhao Y. 2013. Differential lysine acetylation profiles of *Erwinia amylovora* strains revealed by proteomics. J Proteomics 79:60–71. doi:10.1016/j.jprot.2012.12.001. 23234799 PMC4418653

[57] Zhang J, Sprung R, Pei J, Tan X, Kim S, Zhu H, Liu CF, Grishin NV, Zhao Y. 2009. Lysine acetylation is a highly abundant and evolutionarily conserved modification in *Escherichia coli*. Mol Cell Proteomics 8:215–225. doi:10.1074/mcp.M800187-MCP200. 18723842 PMC2634580

[58] Ren J, Sang Y, Tan Y, Tao J, Ni J, Liu S, Fan X, Zhao W, Lu J, Wu W, Yao Y-F. 2016. Acetylation of lysine 201 inhibits the DNA-binding ability of PhoP to regulate *Salmonella* virulence. PLoS Pathog 12:e1005458. doi:10.1371/journal.ppat.1005458. 26943369 PMC4778762

[59] Sang Y, Ren J, Qin R, Liu S, Cui Z, Cheng S, Liu X, Lu J, Tao J, Yao YF. 2017. Acetylation regulating protein stability and DNA-binding ability of HilD, thus modulating *Salmonella* Typhimurium virulence. J Infect Dis 216:1018–1026. doi:10.1093/infdis/jix102. 28329249

[60] Zhou D, Tong Z, Song Y, Han Y, Pei D, Pang X, Zhai J, Li M, Cui B, Qi Z, Jin L, Dai R, Du Z, Wang J, Guo Z, Wang J, Huang P, Yang R. 2004. Genetics of metabolic variations between *Yersinia pestis* biovars and the proposal of a new biovar, microtus. J Bacteriol 186:5147–5152. doi:10.1128/JB.186.15.5147-5152.2004. 15262951 PMC451627

[61] Straley SC, Bowmer WS. 1986. Virulence genes regulated at the transcriptional level by Ca^2+^ in *Yersinia pestis* include structural genes for outer membrane proteins. Infect Immun 51:445–454. doi:10.1128/iai.51.2.445-454.1986. 3002984 PMC262351

[62] Huang da W, Sherman BT, Lempicki RA. 2009. Systematic and integrative analysis of large gene lists using DAVID bioinformatics resources. Nat Protoc 4:44–57. doi:10.1038/nprot.2008.211. 19131956

[63] Philippe N, Alcaraz JP, Coursange E, Geiselmann J, Schneider D. 2004. Improvement of pCVD442, a suicide plasmid for gene allele exchange in bacteria. Plasmid 51:246–255. doi:10.1016/j.plasmid.2004.02.003. 15109831

[64] Chen Y, Song K, Chen X, Li Y, Lv R, Zhang Q, Cui Y, Bi Y, Han Y, Tan Y, Du Z, Yang R, Qi Z, Song Y. 2022. Attenuation of *Yersinia pestis fyuA* mutants caused by iron uptake inhibition and decreased survivability in macrophages. Front Cell Infect Microbiol 12:874773. doi:10.3389/fcimb.2022.874773. 35601093 PMC9114763

[65] Andrews GP, Heath DG, Anderson GW, Jr, Welkos SL, Friedlander AM. 1996. Fraction 1 capsular antigen (F1) purification from *Yersinia pestis* CO92 and from an *Escherichia coli* recombinant strain and efficacy against lethal plague challenge. Infect Immun 64:2180–2187. doi:10.1128/iai.64.6.2180-2187.1996. 8675324 PMC174053

[66] Fang N, Gao H, Wang L, Qu S, Zhang YQ, Yang RF, Zhou DS. 2013. Optimized methods for biofilm analysis in *Yersinia pestis*. Biomed Environ Sci 26:408–411. doi:10.3967/0895-3988.2013.05.012. 23611136

[67] Brown WF. 1964. Variance estimation in the Reed-Muench fifty per cent end-point determination. Am J Hyg 79:37–46. doi:10.1093/oxfordjournals.aje.a120362. 14114354

