# Supplementary material for: Comparative Lysine Acetylome Analysis of Y. pestis YfiQ/CobB Mutants Reveals that Acetylation of SlyA Lys73 Significantly Promotes Biofilm Formation of Y. pestis
Source: Microbiol Spectr. 2023 Jul 17;11(4):e00460-23. doi: 10.1128/spectrum.00460-23 (PMC10433856; doi:10.1128/spectrum.00460-23)
Supplement: Supplemental file 1 — Tables S1, S4, and S5. Download spectrum.00460-23-s0001.docx, DOCX file, 0.02 MB [file spectrum.00460-23-s0001.docx]

**Supplemental Table S1 The numbers of acetylated peptides and proteins detected in this study**

|  | **Replicate 1** | **Replicate 2** | **Replicate3** | **all** | **detected>2 times** | **detected>3 times** |
| --- | --- | --- | --- | --- | --- | --- |
| **acK peptides** |  |  |  |  |  |  |
| **Fv** | 2142 | 2987 | 2811 | 3871 | 2598 | 1471 |
| **Mh** | 2335 | 3834 | 3548 | 4948 | 3066 | 1703 |
| **acK proteins** |  |  |  |  |  |  |
| **Fv** | 775 | 932 | 908 | 1109 | 869 | 637 |
| **Mh** | 727 | 1146 | 1068 | 1311 | 966 | 664 |

**Supplemental Table S4 LD_50_ values of *slyA*_K73Q_ mutant in BALB/c mice via *i.v.* and *s.c*. challenges.**

| Strains | *i.v.* challenged (CFU) | *s.c*. challenged (CFU) |
| --- | --- | --- |
| WT | 1.9 | 3 |
| *slyA*_K73Q_ | 1.6 | 2.65 |

Note: LD_50_ value of wide-type strain is based on the results of our laboratory's previous studies[1]. Five groups of 6 to 8 week female BALB/c mice (n=6 per group) were *i.v.* challenged via caudal vein or *s.c.* challenged at inguina with different doses of each strain.

**Supplemental Table S5 Oligonucleotide probe and primers used in this study**

| Primers | Sequences (5’→3’) |
| --- | --- |
| **Construction of mutants** | |
| Original sequence of SlyA( K73) | TGGATCAACTGGAGGAAAAAGGTTTAATCACACGGCAT |
| SlyA(K73R)-F | GGAACGAGGTTTAATCACACGGCATAC |
| SlyA(K73R)-R | GTATGCCGTGTGATTAAACCTCGTTCCTCCAGTTGATCCAAGGTTC |
| SlyA(K73Q)-F | GGAACAGGGTTTAATCACACGGCATAC |
| SlyA(K73Q)-R | GTATGCCGTGTGATTAAACCCTGTTCCTCCAGTTGATCCAAGGTTC |
| **Expression of SlyA protein** | |
| SlyA-EXP-F | cgcggatccTTGGAATCGACATTAGGATC(Bam HI) |
| SlyA-EXP-R | acgcgtcgacTTACTTAGTTTGTAATTG(Sal I) |
| **EMSA** | |
| psaE-EMSA-F | CCTGTTTGTCCTGCTGATCC |
| psaE-EMSA-R | GACTCATTTGCCCTCACCTC |
| hmsT-EMSA-F | GCCCAGTACAGGTAACAAGG |
| hmsT-EMSA-R | CTGATCGTAGGAGTGGCTATTC |
| slyA-EMSA-F | CGTTCGTTACTCTGCCCATC |
| slyA-EMSA-R | TTGTGATTGCTCTGGTGGTAAAC |
| **Construction of *slyA(K73Q)* strain and the trans-complemented strain** | |
| pDS-*slyA*-F | agaggtaccGCATGCCACCTGACGGAGTCAAACGT |
| pDS-*slyA*-R | ttcccgggaGAGCTCCCAATAGCGGCAGCACAATA |
| pDS132-F | TGAACGGCAGGTATATGTG |
| pDS132-R | AACAAGCCAGGGATGTAACG |
| Seq-*slyA*-F | TCTGGCTATGTCGAGGGGAA |
| Seq-*slyA*-R | GAACCGTACTGATGGTGTGC |
| *slyA*-com-F | GATCGATATCGCTCAGTTGCCGCCTTC |
| *slyA*-com-R | GATCGGATCCCTGCTGTGAATAAAGTCTTTGAAC |

ote: The codon underlined is the site to be mutated, AAA is the lysine codon, CGA is the arginine codon and CAG is the glutamine codon.

1. Yang F, Ke Y, Tan Y, Bi Y, Shi Q, Yang H, Qiu J, Wang X, Guo Z, Ling H *et al*: **Cell membrane is impaired, accompanied by enhanced type III secretion system expression in Yersinia pestis deficient in RovA regulator**. *PLoS One* 2010, **5**(9).
