# Supplementary material for: Comparative Lysine Acetylome Analysis of Y. pestis YfiQ/CobB Mutants Reveals that Acetylation of SlyA Lys73 Significantly Promotes Biofilm Formation of Y. pestis
Source: Microbiol Spectr. 2023 Jul 17;11(4):e00460-23. doi: 10.1128/spectrum.00460-23 (PMC10433856; doi:10.1128/spectrum.00460-23)
Supplement: Supplemental file 3 — Fig. S1-S4.. Download spectrum.00460-23-s0003.docx, DOCX file, 7.2 MB [file spectrum.00460-23-s0003.docx]

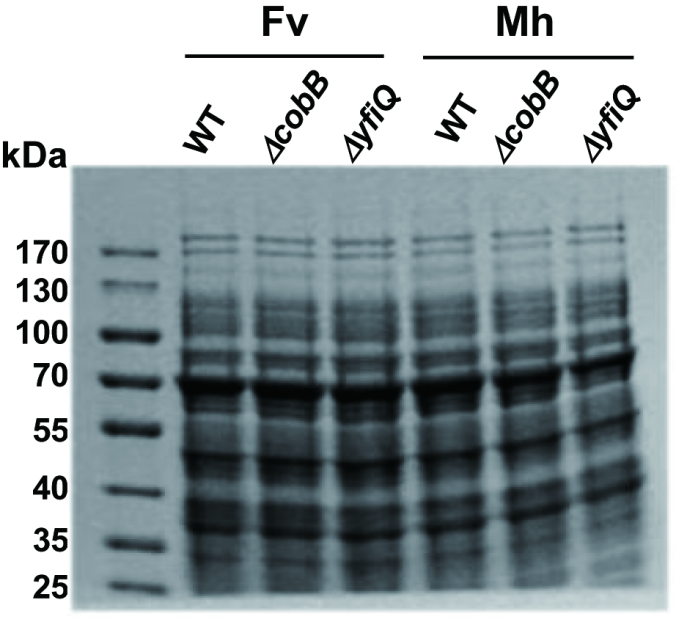


**Supplemental Fig. S1 Protein expression in WT strain and the two mutants cultured under the Fv or Mh condition**. 40 μg of each protein sample extracted from the WT or the mutant strains was sampled and 4-15% SDS-PAGE gradient gel was used. The gel was stained with Coomassi bright blue.

**
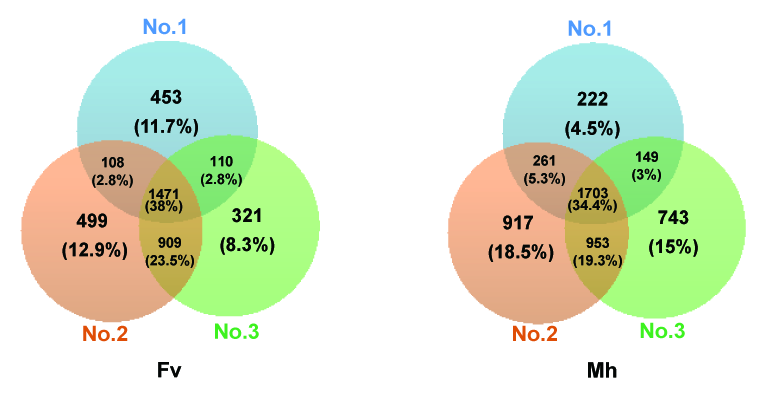
**

**Supplemental Fig. S2 Venn diagram of the acetylated peptides detected in each replicate sample of *Y. pestis* cultured under the Fv condition or Mh condition**.


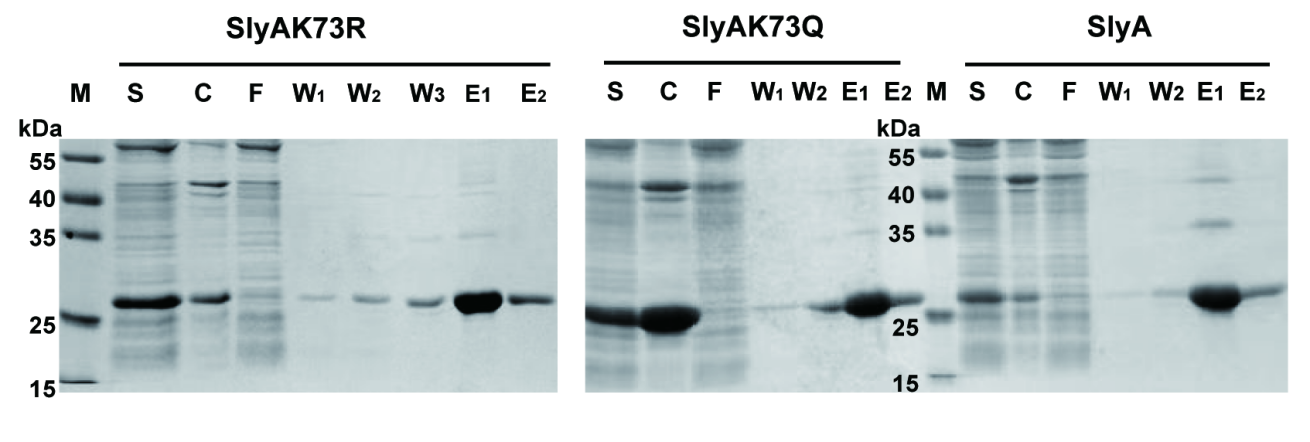


**Supplemental Fig. S3 Electrophoresis of purification process of SlyA and its point mutant proteins.**

M: Markers S: Supernatant C: Cell debris F: Flow W1: Wash 1（20 mM imidazole） W2: Wash 2（40 mM imidazole） W3: Wash 3（60 mM imidazole） E1: Elution 1 E2: Elution 2


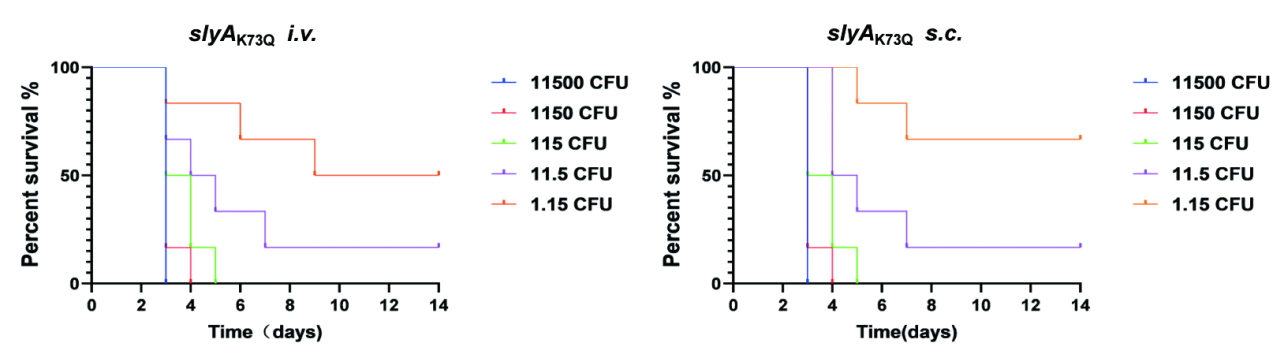


**Supplemental Fig. S4**  **Virulence analysis of the *slyA*_K73Q_ mutant in a mouse model.** Five groups of 6 to 8 week female BALB/c mice (n=6 per group) were challenged with bacterial suspensions of the *slyA*_K73Q_ mutant in PBS at the indicated concentrations via the *i.v.* (A) or *s.c.* (B) routes of infection, and the survival rates were plotted against the days post infection.
